# Degradation and mineralization of 4-tert-butylphenol in water using Fe-doped TiO_2_ catalysts

**DOI:** 10.1038/s41598-019-55775-7

**Published:** 2019-12-17

**Authors:** Ardak Makhatova, Gaukhar Ulykbanova, Shynggys Sadyk, Kali Sarsenbay, Timur Sh. Atabaev, Vassilis J. Inglezakis, Stavros G. Poulopoulos

**Affiliations:** 1 The Environment & Resource Efficiency Cluster (EREC), Nazarbayev University, 010000 Nur-Sultan, Kazakhstan; 2 Environmental Science & Technology Group (ESTg), Chemical and Materials Engineering Department, School of Engineering and Digital Sciences, Nazarbayev University, 010000 Nur-Sultan, Kazakhstan; 3 Department of Chemistry, School of Sciences and Humanities, Nazarbayev University, 010000 Nur-Sultan, Kazakhstan

**Keywords:** Materials science, Photocatalysis, Chemical engineering

## Abstract

In the present work, the photocatalytic degradation and mineralization of 4-tert-butylphenol in water was studied using Fe-doped TiO_2_ nanoparticles under UV light irradiation. Fe-doped TiO_2_ catalysts (0.5, 1, 2 and 4 wt.%) were prepared using wet impregnation and characterized via SEM/EDS, XRD, XRF and TEM, while their photocatalytic activity and stability was attended via total organic carbon, 4-tert-butyl phenol, acetic acid, formic acid and leached iron concentrations measurements. The effect of H_2_O_2_ addition was also examined. The 4% Fe/TiO_2_ demonstrated the highest photocatalytic efficiency in terms of total organic carbon removal (86%). The application of UV/H_2_O_2_ resulted in 31% total organic carbon removal and 100% 4-t-butylphenol conversion, however combining Fe/TiO_2_ catalysts with H_2_O_2_ under UV irradiation did not improve the photocatalytic performance. Increasing the content of iron on the catalyst from 0.5 to 4% considerably decreased the intermediates formed and increased the production of carbon dioxide. The photocatalytic degradation of 4-tert-butylphenol followed pseudo-second order kinetics. Leaching of iron was observed mainly in the case of 4% Fe/TiO_2_, but it was considered negligible taking into account the iron load on catalysts. The electric energy per order was found in the range of 28–147 kWh/m^3^/order and increased with increasing the iron content of the catalyst.

## Introduction

Extensive industrialization in combination with urbanization and overpopulation result in the generation of large amounts of wastewaters. Various substances contained in wastewaters are toxic to plants, animals and people, and pose a threat to environment and human health^1^. For instance, 1,4-dioxane, a probable human carcinogen, can be found in wastewaters released during the manufacture of personal care products, drugs, pesticides, dyes etc.^2^, while pharmaceuticals and cosmetics from untreated domestic wastewaters were detected in the urban river across the megacity of Shanghai^3^. The proper treatment of wastewaters is therefore important for sustainable development and people well-being.

Endocrine disrupting compounds (EDCs) constitute a serious concern for water quality, since they may affect endocrine system even at very low concentrations^4^. 4-tert-butylphenol (4-t-BP) is an alkylphenol and one of the EDCs that combine poor biological degradability^5^ and high estrogenic effect^6–8^. 4-t-BP is widely used as raw material for the production of phosphate esters, oil field chemicals, fragrances, demulsifiers^9^, polymerization inhibitors and stabilizing agents in the chemical industry^5^. It can be spread in the aquatic environment including sea and river waters and sediments. It has been detected in effluent samples from sewage and wastewater treatment plants^10^. The removal of 4-tert-butylphenol from the aquatic media is thus essential for the protection of the environment^11^ and human health^5^, since its persistence in the environment^12^ in combination with its acute and chronic toxicity^13^, and its estrogenic activity^14^ as well, classify 4-t-BP among the pollutants of emerging concern. Biological processes have been proved time consuming and inefficient in the degradation of 4-t-BP^10,11^.

Heterogeneous photocatalysis has the potential to decompose toxic pollutants in water and has been applied successfully for the elimination of many hazardous organic compounds. However, the photocatalytic degradation of 4-tert-butylphenol has been scarcely investigated^12^. Moreover, the path of 4-t-BP photodegradation in aqueous solutions has not been understood^15^.

Heterogeneous photocatalysis belongs to the Advanced Oxidation Processes (AOPs), which have been effectively used for the removal of recalcitrant organic compounds^16^. The efficiency of AOPs depends on the formation of highly active free radicals^17^, which are atoms or molecules with one or several unpaired electrons^18^. Among various oxidative agents, hydroxyl radicals play a central role in AOPs for effluent treatment^19^. They are used as highly reactive species in UV/H_2_O_2_, photo-Fenton/Fenton-like systems, photocatalytic oxidation, and others^20^.

Titanium dioxide has high stability to light irradiation, relatively high activity, low cost and non-toxicity among many semiconductor photocatalysts^21^. It can exist in three crystallographic forms, namely anatase, rutile and brookite^22^. Degussa P25 is a mixture of 70% anatase and 30% rutile, and improved degradation efficiencies have been reported with its use compared to other photocatalysts^21^. TiO_2_-mediated photocatalysis has been widely used in the wastewater treatment. The benefits of this process include work under ambient conditions, the absence of mass transfer limitations when nanoparticles are used as photocatalysts, highly oxidizing photogenerated holes, cheap and readily available forms of TiO_2_, and the possibility of using solar irradiation^23^. TiO_2_ can oxidize various organic substances ultimately to CO_2_ and H_2_O^24^.

An increasing number of studies is focused on improving the efficiency of photocatalysts by either expanding the absorption spectrum to visible light or slowing down the recombination rate between electrons and holes and increasing thus the efficiency of interfacial charge transfer. This is attempted by doping TiO_2_ with metals, metal ions, non-metal atoms, and semiconducting oxides^25^.

Wang *et al*.^26^ used templates of silver oxide octahedra and titanium tetrafluoride as precursor to prepare hollow octahedra of silver-modified titanium dioxide. The prepared catalysts showed enhanced photocatalytic performance as a result of fast electron transfer between titanium dioxide and silver nanoparticles. Moreover, Wang *et al*.^27^ applied a combination of hydrothermal and photodeposition techniques to prepare a novel Ag/F-TiO_2_ photocatalyst, which exhibited improved photocatalytic activity compared to the reference TiO_2_, F-TiO_2_, and Ag/TiO_2_ catalysts.

The dehydrogenative methane coupling to ethane was conducted with increased energy efficiency on a gold-modified TiO_2_ photocatalyst, which was synthesized via the photodegradation of gold nanoparticles on the polar {001} facet of titanium dioxide in the anatase form^28^. Similarly, the efficiency of solar-to-hydrogen conversion was increased by a factor of 64 via introducing gold nanoparticles on the {001} facets of nanosheets of anatase^29^.

A photodeposition technique was also applied by Gao *et al*.^30^ to prepare Sn/TiO_2_ photocatalysts with considerably enhanced performance in H_2_ generation compared to that of the titanium dioxide base catalyst.

Huang *et al*.^31^ achieved a 0.56% quantum efficiency in the carbon dioxide reduction to form methane under visible light illumination by means of a novel triad photocatalyst based on a mononuclear C_5_H_5_-RuH complex oxo-bridged TiO_2_ hybrid.

A graphene–titanium dioxide catalyst with magnetic properties exhibited higher removals than the base titanium dioxide during the treatment of Methylene Blue and tetrabromobisphenol A in water^32^.

The type-II nanostructured TiO_2_@Ta_2_O_x_N_y_ nanorods photoanodes were shown to achieve a 12 fold-enhanced photoelectrocatalytic water splitting efficiency under solar light irradiation as well as a solar-to-chemical energy conversion efficiency of ca. 1.49% at 1.23 V vs RHE^33^.

A novel Bi-BiOI/GR composite photocatalyst prepared solvothermally showed improved performance in the oxidation of nitric oxide under visible light irradiation in relation with the pure BiOI photocatalyst^34^.

The photocatalytic activity of Fe-doped TiO_2_ nanoparticles has been examined in a number of studies^35–43^. Choi *et al*.^35^ studied the effect of doping 21 metal ions into TiO_2_ and found that doping with Fe^3+^ at 0.1–0.5% significantly increased the photoreactivity for both oxidation and reduction. Anwar *et al*.^41^ synthesized Fe-doped TiO_2_ catalyst with the concentration of 6 wt.% iron through a sol-gel method, and the Fe/TiO_2_ catalyst efficiently degraded methylene blue dye under UV and visible light irradiation.

The presence of iron particles can favourably affect the photocatalytic activity, which may be due to the role of iron particles acting as h^+^/e^−^ traps, thereby inhibiting the recombination rate and enhancing the photocatalytic activity^44^. However, further study of Fe-doped TiO_2_ catalysts is required, and 4-t-BP, a dangerous endocrine disruptor, is an ideal target compound in water as it has not been studied previously.

In this work, Fe-doped TiO_2_ catalysts with different iron content (Fe/Ti weight ratio percentage = 0.5%, 1%, 2% and 4%) were prepared and used to eliminate 4-tert-butylphenol in water under ultraviolet light irradiation. The effect of adding hydrogen peroxide in the photocatalytic system was also investigated. Although hundreds or even thousands of studies exist for target compounds in water like phenol, only a very limited number of works have been devoted to 4-tert-butylphenol, which is an endocrine disruptor and a serious emerging environmental concern. To the best of our knowledge, a study on the photocatalytic mineralization of 4-tert-butylphenol in water using Fe-doped TiO_2_ catalysts has not been conducted before.

## Results and Discussion

### Characterization of catalysts

**Figure 1:**
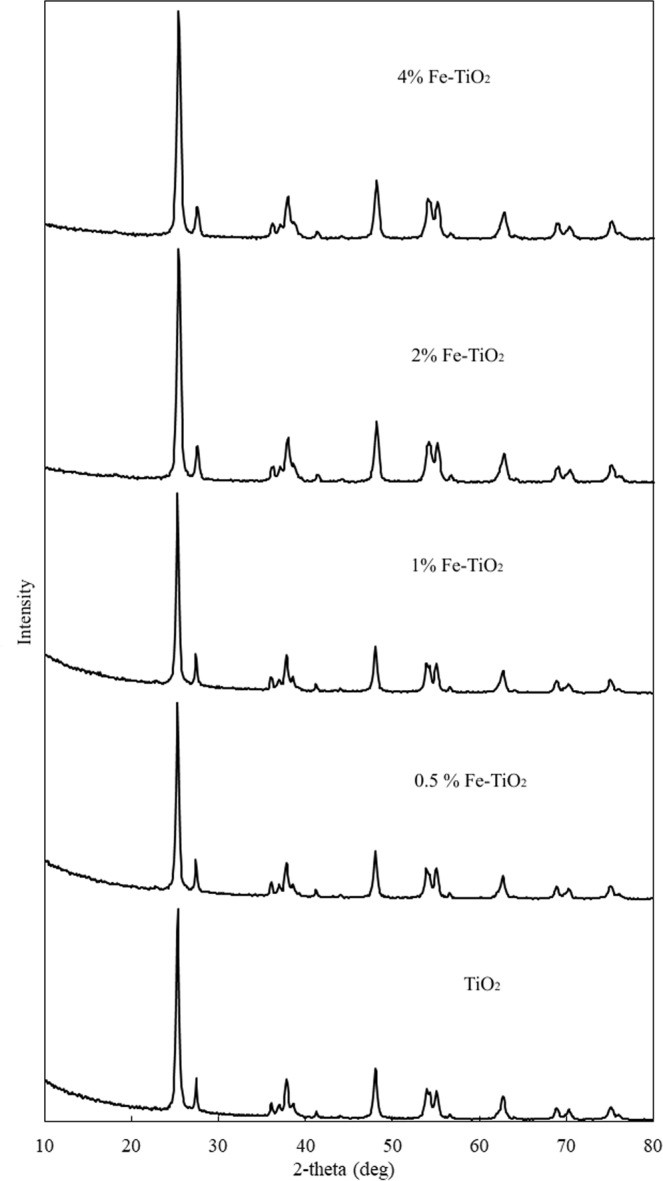
X-ray diffraction patterns of Fe-doped and undoped TiO_2_ catalysts.

The XRD patterns of Fe-doped and undoped TiO_2_ catalysts are shown in Fig. 1. The diffraction peaks at 2θ = 25.3°, 37.8°, 48.0°, 54.0°, and 55.1° are attributed to the anatase phase of TiO_2_ (ICDD No. 86–1048, 86-1157). The diffraction peak at 2θ = 27.4° is attributed to the rutile phase of TiO_2_^45^. The X-ray diffraction pattern of anatase has a major peak at 2θ = 25.3°. The XRD patterns for all synthesized catalysts were similar to the one for the base TiO_2_ catalyst. No crystalline iron-related phase was observed, which has been also reported in similar studies previously^42,46,47^. This result can be explained by the fact that crystalline forms of Fe were not formed on the material or that particles of amorphous iron oxides were too small on the surface of TiO_2_ particles^47^. In addition, the peak associated with iron cannot be observed in the XRD spectra when all iron ions are either incorporated into the TiO_2_ structures replacing titanium ions or are at interstitial site due to similar ionic radii (Ti (0.68 Å) and Fe (0.64 Å))^42,46^.

The TiO_2_ crystallite sizes calculated by the Scherrer equation on the anatase diffraction peak (2θ = 25.3°) are listed in Table 1. The average particle size of Fe/TiO_2_ catalysts was found in the range of 17-21 nm, which is agreement with the TiO_2_ specifications.

**Table 1:** Calculated crystallite sizes of undoped and Fe-doped catalysts.

Sample	Crystallite size, nm
TiO_2_	20.56
0.5% Fe/TiO_2_	18.68
1% Fe/TiO_2_	21.03
2% Fe/TiO_2_	19.31
4% Fe/TiO_2_	17.24

XRF analysis revealed the iron loading of the synthesized 4% Fe/TiO_2_ catalyst as Fe_2_O_3_/TiO_2_ = 3.74%. The results of XRF analysis are presented in Table 2. The chemical composition of the synthesized catalyst revealed that it was consisted of TiO_2_ (96.174%), Fe_2_O_3_ (3.597%) and CaO (0.078%), SiO_2_ (0.011%), Cl (0.139%) and the ratio of Fe_2_O_3_/TiO_2_ was 3.597%/96.174% = 3.74%. These results confirmed the existence of iron as dopant in the sample and showed that iron was in its oxide form.

**Table 2:** XRF analysis of 4% Fe/TiO_2_ catalyst.

Compound	Concentration (%)
SiO_2_	0.011
Cl	0.139
CaO	0.078
TiO_2_	96.174
F_2_O_3_	3.597

**Figure 2:**
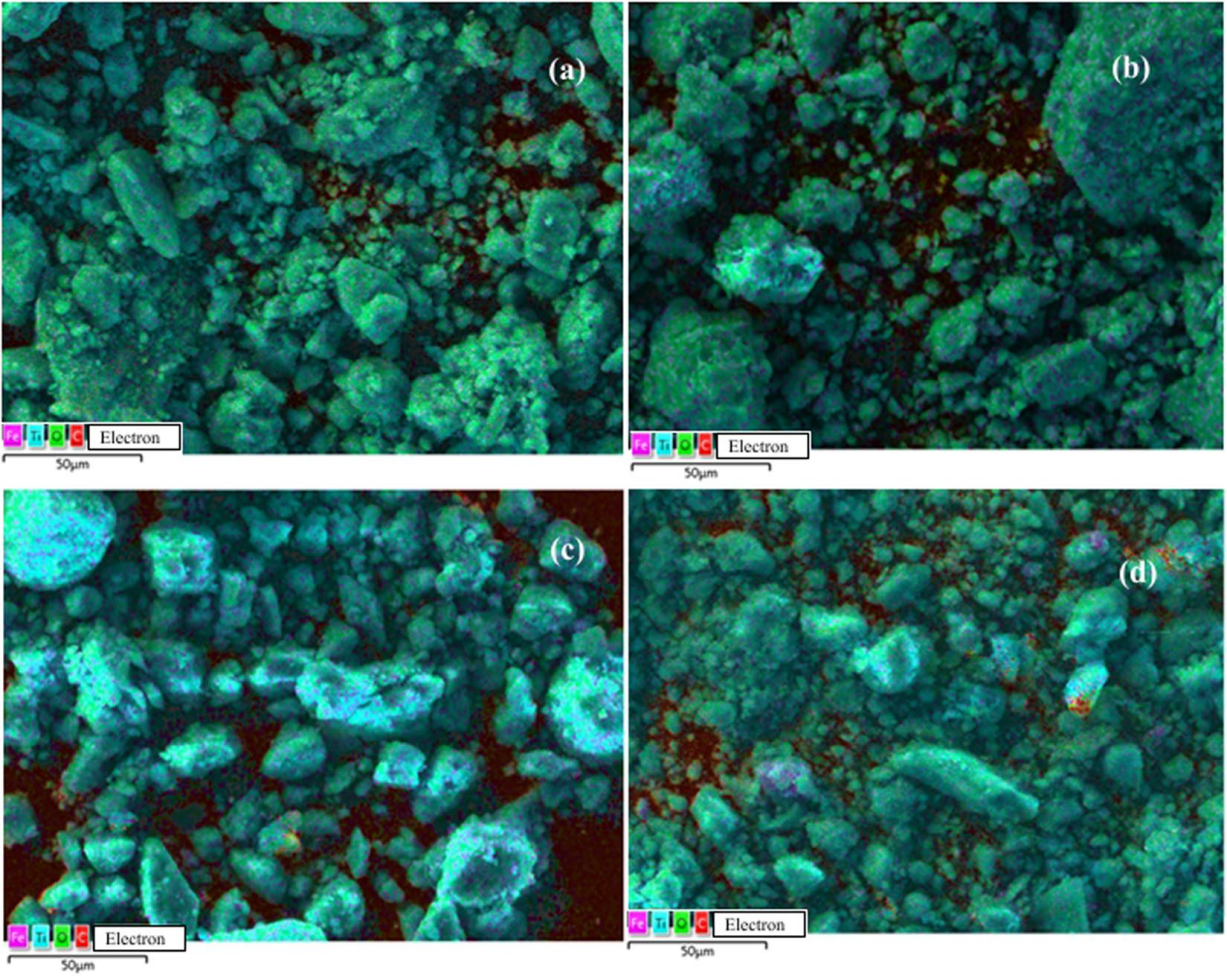
SEM/EDS analysis of (**a**) 0.5% Fe/TiO_2_; (**b**) 1% Fe/TiO_2_; (**c**) 2% Fe/TiO_2_; (**d**) 4% Fe/TiO_2_.

**Figure 3:**
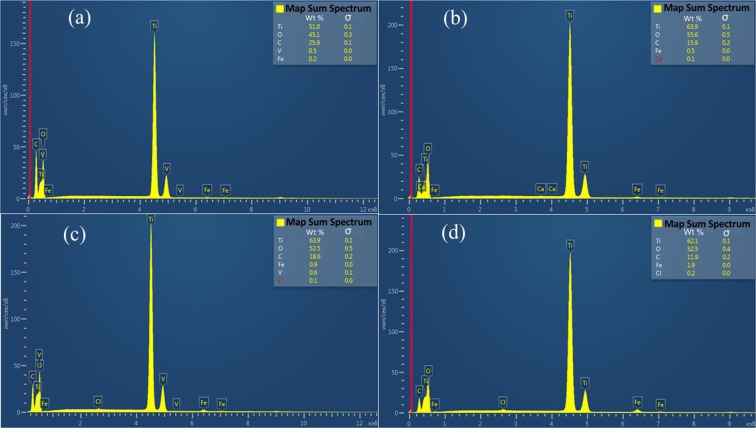
SEM/EDS spectrum of (**a**) 0.5% Fe/TiO_2_; (**b**) 1% Fe/TiO_2_; (**c**) 2% Fe/TiO_2_; (**d**) 4% Fe/TiO_2_.

Elemental mappings (SEM/EDS) for all Fe/TiO_2_ catalysts are presented in Figs. 2 and 3. SEM/EDS provided information on the elements present and their quantities. A well-distributed iron phase on the surface of TiO_2_ was observed in all cases. No differences among catalysts were observed in EDS mappings. SEM analysis showed that the surface morphology of Fe/TiO_2_ catalysts used remained unaltered after the photocatalytic reaction. It can be observed that particles tended to agglomerate.

**Figure 4:**
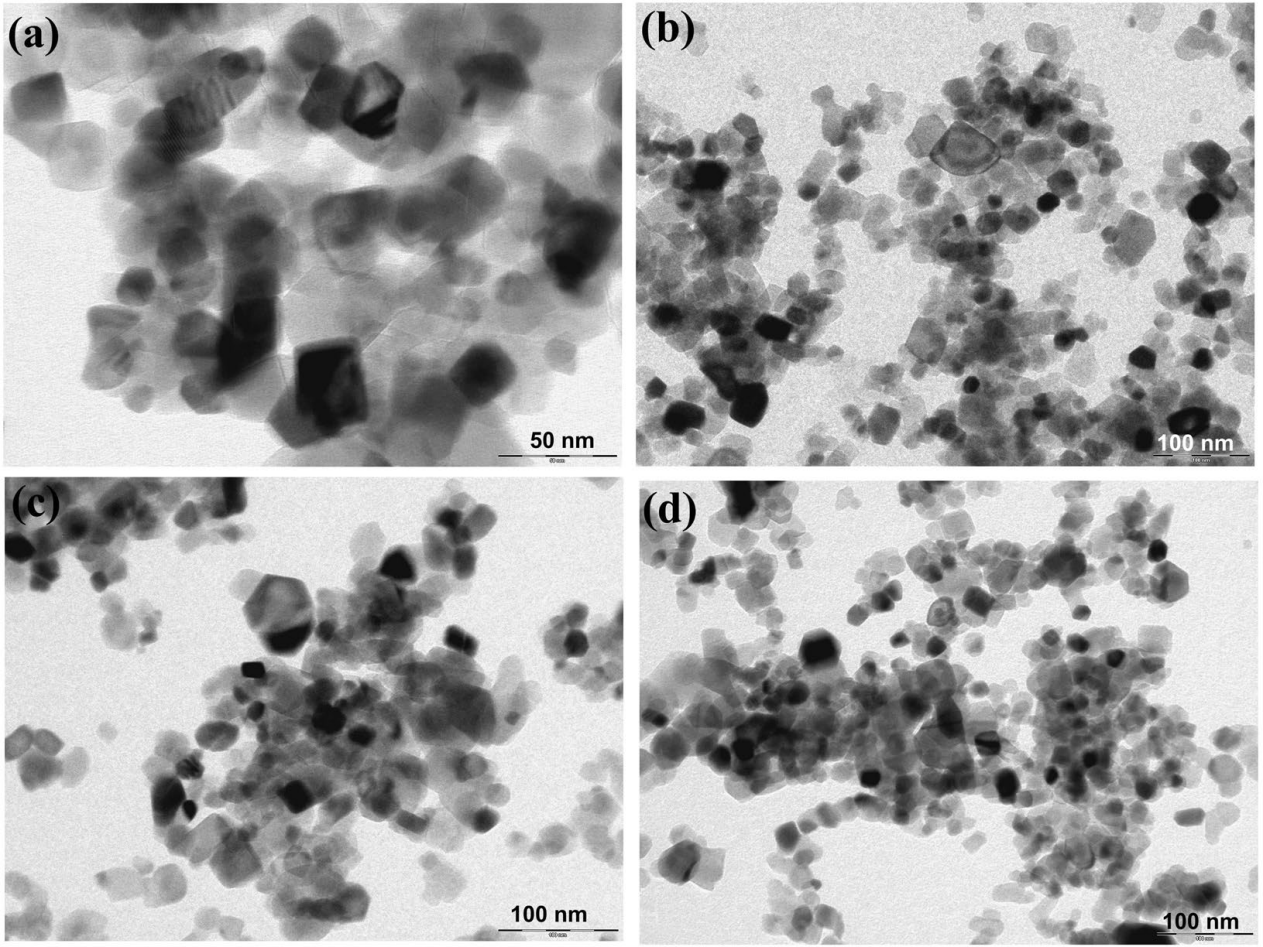
TEM analysis of (**a**) 0.5% Fe/TiO_2_; (**b**) 1% Fe/TiO_2_; (**c**) 2% Fe/TiO_2_; (**d**) 4% Fe/TiO_2_.

TEM images of the catalyst are shown in Fig. 4. It was found that the particle size was around 20 nm.

**Figure 5:**
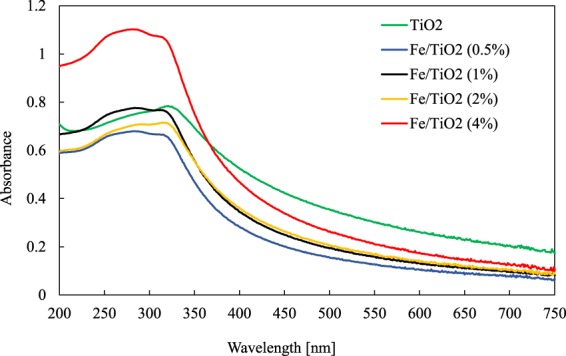
The UV-Vis spectra of TiO_2_ and Fe-TiO_2_ with different iron doping concentrations.

The optical properties of fresh catalysts were measured using a UV-Vis spectroscopy in the wavelength range of 200 to 750 nm (Fig. 5). The prepared Fe/TiO_2_ catalysts had relatively the same absorption as the base TiO_2_, and only the 4% Fe/TiO_2_ showed a significantly higher absorption in the range of 200–365 nm.

The shift of the absorption range of 4% Fe/TiO_2_ can be explained by the transition of charge transfer between the d-electrons of the iron ion and the conduction or valence band of TiO_2_^48^.

### Photocatalytic treatment of 4-t-BP

Titanium dioxide semiconductor absorbs ultraviolet light and generates hydroxyl radicals according to the following reactions^49^:

1 $$Ti{O}_{2}+h\nu \to {e}_{cb}^{-}+h{v}_{vb}^{+}$$

2 $${e}_{cb}^{-}+{O}_{2}\to {O}_{2}^{-\bullet }$$

3 $$h{v}_{vb}^{+}+{H}_{2}O\to O{H}^{\bullet }+{H}^{+}$$

Oxidative degradation of organic compounds can occur through their reactions with hydroxyl and peroxide radicals, valence band holes, and reductive splitting through their reactions with electrons^23^.

The presence of iron particles can favourably affect the photocatalytic activity, which may be due to the role of iron particles acting as h^+^/e^−^ traps, thereby inhibiting the recombination rate and enhancing the photocatalytic activity^44^. Equations (4–8) show the detailed reaction steps^50,51^:

4 $$F{e}^{3+}+{e}_{cb}^{-}\to F{e}^{2+}$$

5 $$F{e}^{2+}+{O}_{2(ads)}\to F{e}^{3+}+{O}_{2}^{-\bullet }$$

6 $$F{e}^{2+}+T{i}^{4+}\to F{e}^{3+}+T{i}^{3+}$$

7 $$F{e}^{3+}+h{v}_{vb}^{+}\to F{e}^{4+}$$

8 $$F{e}^{4+}+O{H}^{-}\to F{e}^{3+}+H{O}^{\bullet }$$

However, when the concentration of iron is high, iron ions can also act as recombination centres for h^+^/e^−^ pairs, according to Eqs. (9) and (10)^50^:

9 $$F{e}^{3+}+{e}_{cb}^{-}\to F{e}^{2+}$$

10 $$F{e}^{2+}+h{v}_{vb}^{+}\to F{e}^{3+}$$

In addition, an excess of deposited iron on TiO_2_ can form Fe (OH)^2+^ particles with higher light absorption in the range of 290–400 nm compared to TiO_2_. The competition in the absorption of photons subtracts the photon to TiO_2_ and can reduce thus the photocatalytic activity of Fe/TiO_2_ catalysts^52^.

The overall reaction of photocatalytic degradation of 4-tert-butylphenol can be represented by Eq. (11):

11 $$10\,{C}_{10}{H}_{14}O+95\,{O}_{2}\to 100\,C{O}_{2}+7{H}_{2}O$$

**Figure 6:**
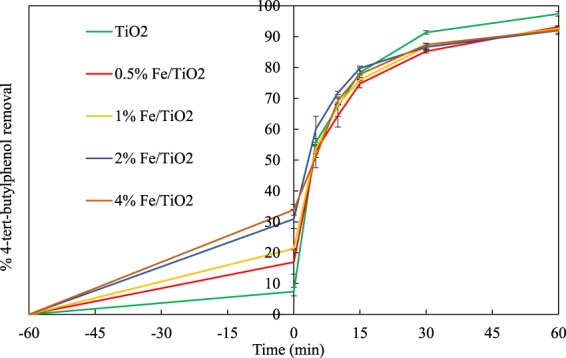
4-t-BP removals vs. time during its photocatalytic degradation by UV/TiO_2_ and UV/Fe-TiO_2_ processes.

The initial pH of the solution was around 6.3, and it decreased to about 3.8 at the end of the experiments. 4-tert-butylphenol was effectively removed by all studied catalysts, as it is shown in Fig. 6. The maximum removal of 4-t-BP was observed with the base TiO_2_ catalyst reaching 97%. All Fe-doped TiO_2_ catalysts led to 92–93% removal of 4-t-BP even though they exhibited superior adsorption capacity.

For the estimation of adsorption capacity, 0.25 g of each catalyst was added to 0.25 L of aqueous solution containing 4-tert-butylphenol (30 mg L^−1^). The solution was then stirred for 1 hour in the dark, before UV irradiation, so that the system reached adsorption equilibrium. The equilibrium adsorption capacity was calculated according to Eq. (12):

12 $${q}_{e}=\frac{({C}_{0}-{C}_{e})V}{m}$$

where:

*q*_*e*_ = the adsorption capacity of the catalyst at the equilibrium time, mg g^−1^

*C*_0_ = the initial concentration of 4-t-BP in the solution, mg L^−1^

*C*_*e*_ = the equilibrium concentration of 4-t-BP in the solution, mg L^−1^

*V* = the volume of the solution, L

*m* = the mass of the catalyst, g.

**Figure 7:**
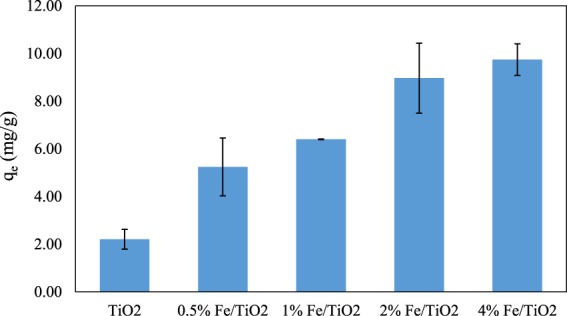
The adsorption capacity (mg g^−1^) of each catalyst at the equilibrium time.

The results obtained are presented in Fig. 7. Increasing the iron content of the catalyst led to increasing q_e_ values, which however were low compared to values reported elsewhere^53^.

Adsorption can be either beneficial or unfavourable to photocatalysis. For example, Lu Lin *et al*.^54^ studied the adsorption and photocatalytic oxidation of ibuprofen using nanocomposites of TiO_2_ nanofibers with boron nitride nanosheets, and they reported that the adsorption of the target compound molecules resulted in increased photocatalytic degradation rates due to better transfer of photogenerated radicals on the catalytic surface in some cases, while for some catalysts the photocatalytic process was adversely affected as a result of the screening of light access to the catalysis by adsorbed molecules.

**Figure 8:**
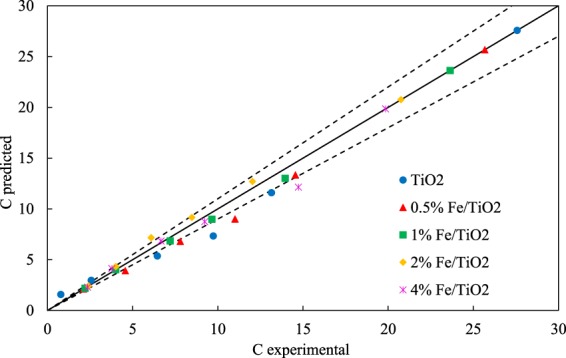
Predicted concentration values of 4-t-BP vs. experimental ones using pseudo-second order kinetics.

To investigate the kinetics of 4-t-BP photocatalytic degradation, a pseudo-first order and a pseudo-second order kinetic model were tested as represented by the following equations, respectively:

13 $$\frac{ln{C}_{t}}{{C}_{o}}=-\,{k}_{1}t$$

14 $$\frac{1}{{C}_{t}}-\frac{1}{{C}_{0}}=-\,{k}_{2}t$$

where *k*_1_ and *k*_2_ are the corresponding reaction rate coefficients. The experimental data were best fitted by the pseudo-second order model as presented in Fig. 8. The reaction rate coefficients calculated are shown in Table 3.

**Table 3:** Reaction rate coefficients considering pseudo-second order kinetics for 4-tert-butylphenol photocatalytic degradation.

Sample	k (L mol^−1^ min^−1^)	R^2^
TiO_2_	0.01	0.9486
0.5% Fe/TiO_2_	0.0072	0.9918
1% Fe/TiO_2_	0.0069	0.9995
2% Fe/TiO_2_	0.0061	0.9947
4% Fe/TiO_2_	0.0064	0.9917

**Figure 9:**
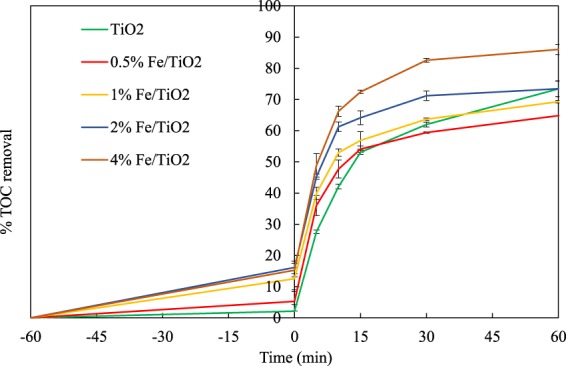
TOC removals vs. time during the photocatalytic degradation of 4-t-BP by UV/TiO_2_ and UV/Fe-TiO_2_ processes.

TOC removals for all catalysts are shown in Fig. 9. After 60 min of UV irradiation, 73% of the initial TOC was eliminated with the base TiO_2_ catalyst. The TOC removal increased with increasing the iron doping content from 0.5% to 4%, resulting in 65% and 86% removal, respectively. The 2% Fe/TiO_2_ catalyst led to the same TOC removal as TiO_2_, whereas 4% Fe/TiO_2_ had higher removal efficiency than TiO_2_. Controversial results are reported in literature regarding the effect of iron doping concentration. Specifically, according to Zhu *et al*.^38^, the 0.40% Fe-TiO_2_ showed a higher photoactivity than both the undoped TiO_2_ and the commercial photocatalyst Degussa P25 under UV irradiation. Much more oxygen vacancies in the crystal lattice and on TiO_2_ surface were introduced by doping with iron. Zhao *et al*.^47^ studied the photocatalytic degradation of 4-nitrophenol using Fe-doped (1, 3, 5 and 8 wt.% Fe) TiO_2_ catalysts. The catalysts with the lowest Fe content (1%) showed a considerably better behaviour than TiO_2_ and the TiO_2_ catalysts with higher Fe contents. On the other hand, Nikolaki *et al*.^43^ showed that the 10% Fe/TiO_2_ catalyst efficiently degraded 1,3-dichloro-2-propanol in water. Anwar *et al*.^41^ synthesized 6 wt.% Fe/TiO_2_ catalyst, which exhibited high performance in the degradation of methylene blue under UV and visible light irradiation.

The higher TOC removals observed compared to 4-t-BP removals can be attributed to the formation of 4-t-BP intermediates, which were more resistant to the photocatalytic process than the parent compound.

In the irradiated solution, there is competition between the parent pollutant and the formed intermediate compounds for oxidizing agents^55^. The base TiO_2_ catalyst seems to have favoured the conversion of 4-t-BP, while the 4% Fe/TiO_2_ catalyst clearly promoted the carbon oxidation to CO_2_.

### Effect of H_2_O_2_ addition

The combination of catalysts and hydrogen peroxide under UV irradiation was also investigated. The purpose was to examine whether the photocatalytic degradation of 4-t-BP could be enhanced as a result of heterogeneous photo-Fenton reaction^47^.

The Fenton method is the oldest and most used chemical AOP, in which the Fenton’s reagent, a mixture of a soluble iron(II) salt (catalyst) and hydrogen peroxide (oxidant), is used to destroy recalcitrant organic compounds^56,57^. The classical Fenton reaction under UV irradiation is called photo-Fenton process (Eq. 15), which enhances the catalytic reduction of Fe^3+^ into Fe^2+^ in H_2_O_2_ aqueous solutions, thereby increasing the generation of •OH radicals^58^:

15 $$F{e}^{3+}+{H}_{2}O+h\nu \to F{e}^{2+}+{H}^{+}+O{H}^{\bullet }$$

The heterogeneous photo-Fenton degradation of 4-t-BP can have a beneficial effect due to an increase in the concentration of generated hydroxyl radicals^47^:

16 $${H}_{2}{O}_{2}+h\nu \to 2H{O}^{\bullet }$$

17 $${H}_{2}{O}_{2}+{e}_{cb}^{-}\to H{O}^{\bullet }+O{H}^{-}$$

18 $${H}_{2}{O}_{2}+F{e}^{2+}\to F{e}^{3+}+H{O}^{\bullet }+O{H}^{-}$$

19 $$RH+O{H}^{\bullet }\to {H}_{2}O+{R}^{\bullet }\to further\,oxidation$$

**Figure 10:**
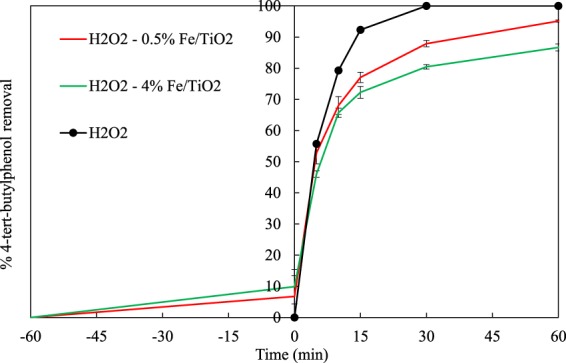
4-t-BP removals vs. time by UV/H_2_O_2_, UV/H_2_O_2_/TiO_2_ and UV/H_2_O_2_/Fe-TiO_2_ processes.

**Figure 11:**
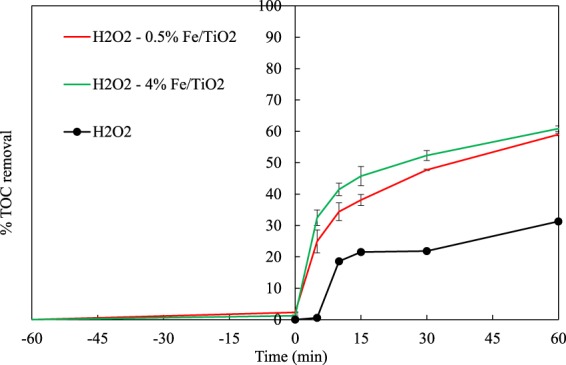
TOC removals vs. time during the 4-t-BP degradation by UV/H_2_O_2_, UV/H_2_O_2_/TiO_2_ and UV/H_2_O_2_/Fe-TiO_2_ processes.

The photochemical treatment by means of UV/H_2_O_2_ was also studied for comparison purposes. 100% 4-t-BP degradation (after only 30 minutes) and 31% TOC removal were obtained, as shown in Figs. 10 and 11, respectively. Although H_2_O_2_ led to complete conversion of 4-t-BP faster than all catalysts tested, it showed much less activity in the oxidation of carbon to CO_2_. In the presence of hydrogen peroxide, the 4-t-BP degradation was adversely affected by increasing the iron content on the catalyst; the 4-t-BP removal decreased from 95% for 0.5% Fe/TiO_2_ catalyst to 87% for 4% Fe/TiO_2_.

Regardless of the Fe doping concentration, both 0.5% and 4% Fe/TiO_2_ catalysts showed final TOC removals in the range of 59–61%.

The results showed that the addition of hydrogen peroxide was not beneficial for the photocatalytic treatment in terms of TOC removal. Previously reported results on the efficiency of combining hydrogen peroxide with heterogeneous catalysts under UV irradiation are contradictory. Some studies reported that this combination increased the efficiency of the process, since UV light was combined with both the oxidant and the photocatalyst^59^, while others claimed that the efficiency of the process was reduced due to the competition for ultraviolet irradiation between the oxidant and the photocatalyst^60^ or due to H_2_O_2_ adsorption on the surface of catalytic particles, which reduced the activity of the catalyst^61^. In addition, it could be suggested that hydrogen peroxide was consumed at the beginning of the process, and the rest of the organic matter was removed by the catalyst^55^.

### Formation of intermediates

Acetic and formic acid were quantified, as they are the last intermediates during the decomposition of organic compounds before the formation of CO_2_ and H_2_O. The formation of acetic acid and formic acid increased with increasing the iron content on the catalyst. The concentrations of these acids increased also with the addition of H_2_O_2_.

The measurements of the concentrations of the parent compound, acetic and formic acid, and of organic carbon, allowed the indirect calculation of the carbon corresponding to the intermediates formed (other than acetic and formic acid) during the photocatalytic degradation of 4-t-BP. Specifically, the concentration of intermediates and CO_2_ at the end of the process can be calculated according to the following material balances expressed in terms of organic carbon (OC), respectively:

20 $$O{C}_{intermediates}=final\,(TO{C}_{overall}-TO{C}_{4-t-BP}-TO{C}_{acids})$$

21 $$O{C}_{C{O}_{2}}=initial\,TO{C}_{overall}-final\,TO{C}_{overall}$$

**Figure 12:**
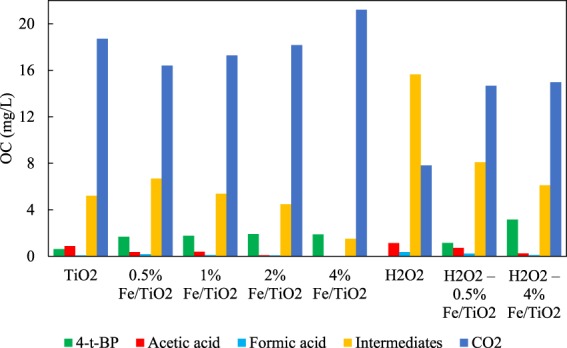
The concentration of 4-t-BP, formic acid, acetic acid, carbon dioxide, and intermediates expressed in mg L^−1^ of organic carbon for each process.

The results obtained are presented in Fig. 12. Although the 4-t-BP conversion was practically the same, the increase in iron content on the catalyst from 0.5% to 4% decreased considerably the carbon of intermediates, increasing in parallel the formation of CO_2_. It is noteworthy that the photochemical degradation via H_2_O_2_ led quickly to 100% removal of 4-t-BP without increasing the formation of intermediates and acids. The use of iron catalysts led again to decreased concentrations of intermediates and acetic and formic acids, while it enhanced the generation of CO_2_.

The path of the photo-degradation of 4-t-BP in aqueous solutions has not been studied in detail. Wu *et al*.^15^ studied the UV photolysis, laser flash photolysis and 4-t-BP oxidation with H_2_O_2_ in water. They detected 4-tert-butylcatechol, 4-tert-butylphenol dimer, 6-tert-butyl-3-methylanisole, benzene-1,4-diol, and 2-nonen-1-ol, 2-decen-1-ol, 2-dodecenal as intermediate products. Wu *et al*.^62^ studied the UV-C direct photolysis, UV/H_2_O_2_ and UV/S_2_O_8_^2−^ degradation of 4-t-BP and reported 2,4-di-tert-butylphenol and 4-tert-butylphenol dimer as by products during the UV/S_2_O_8_^2−^ process.

Xiao *et al*.^12^ synthesized Bi_4_O_5_I_2_ nanoflakes and tested them in the degradation and mineralization of 4-tert-butylphenol in water using visible light. They were not able to detect any intermediates at the end of their experiments (2 hours) and reported only small amounts of isobutyl acetate and butyl acetate as well as very small amount of ethylbenzene after 15 min of photocatalytic treatment.

### Stability of Fe/TiO_2_ catalysts

The stability of the catalysts prepared was examined by determining the iron released in the solution at the end of photocatalytic experiments. Fe leaching was observed for 4% Fe/TiO_2_ during all experiments. Specifically, 0.01 ppm of Fe were detected for 0.5–2% Fe/TiO_2_ catalysts and 0.14 ppm for the 4% Fe/TiO_2_ catalyst. The presence of hydrogen peroxide contributed to iron leaching, since 0.04 ppm of Fe were detected for the 0.5% Fe/TiO_2_ catalyst and 0.40 ppm for the 4% Fe/TiO_2_ catalyst. In the case of 4% Fe/TiO_2_, the iron concentrations measured in the solution corresponded to 0.35% and 1% of the Fe amount on the catalyst in the absence and in the presence of hydrogen peroxide, respectively. So, the extent of Fe leaching can be considered insignificant compared to the total amount of iron deposited on the surface of TiO_2_, and therefore it did not play any role in the performance of the catalysts.

### Energy consumption

An assessment of the energy consumption of the ultraviolet lamp is important because it increases the operating cost of wastewater treatment. The electric energy per order, *E*_*EO*_ (kWh/m^3^/order), is commonly used, which can be estimated using Eq. (22) for a batch reactor^63^:

22 $${E}_{EO}=\frac{P\cdot t\cdot 1000}{V\cdot 60\cdot log({C}_{o}/{C}_{f})}$$

where:

*P* = electrical power of the UV lamp, kW

*t* = irradiation time, min

*V* = the volume of the treated wastewater, L

*C*_*o*_ = the initial concentration of the pollutant, mg L^−1^

*C*_*f*_ = the final concentration of the pollutant, mg L^−1^.

TOC values were used for *C*_*o*_ and *C*_*f*_, and the results obtained are shown in Table 4. The values obtained were in the range 28–147 kWh/m^3^/order. It is obvious that the effect of the process applied on the energy consumed was important. The addition of hydrogen peroxide affected *E*_*EO*_ resulting in higher energy consumptions. Increasing the iron doping of TiO_2_ catalysts decreased the energy consumption. The results are in accordance with previously reported ones. Specifically, Foteinis *et al*.^64^ estimated *E*_*EO*_ values in the range of 4–958 kWh/m^3^/order in the photochemical oxidation of an endocrine disrupting micro-pollutant. The authors reported a high dependence of energy consumption on the process used and the fact that with the addition of small amounts of oxidative agents, the environmental impact can be significantly reduced.

**Table 4:** The consumption of electrical energy per treatment process.

Process	E_EO_, kWh/m^3^/order
UV + 4% Fe/TiO_2_	28
UV + 2% Fe/TiO_2_	42
UV + 1% Fe/TiO_2_	47
UV + 0.5% Fe/TiO_2_	53
UV + TiO_2_	42
UV + H_2_O_2_ + 4% Fe/TiO_2_	59
UV + H_2_O_2_ + 0.5% Fe/TiO_2_	62
UV + H_2_O_2_	147

## Materials and Methods

### Reagents

4-tert-butylphenol (99%) used as target pollutant, and titanium (IV) oxide (P25, nanopowder, 21 nm primary particle size, ≥99.5%) used as the base photocatalyst, were supplied by Sigma-Aldrich. The ultrapure water used in all experiments was obtained by means of Direct-Q 3UV water purification system. Iron (II) chloride (98%) purchased from Sigma-Aldrich was used for doping the TiO_2_ photocatalyst. Hydrogen peroxide solution (37.6% w/w) received from Skat-Reactiv company was used as source of hydroxyl radicals. All chemical reagents were used without further purification.

### Preparation of 4-tert-butylphenol solution

1000 mL stock solution of 4-tert-butylphenol with a concentration of 300 ppm was prepared in ultrapure water. The solutions used were prepared by further suitable dilution of the stock solution to the desired concentration of 30 ppm of 4-t-BP. The initial 4-t-BP concentration was measured via High-Pressure Liquid Chromatography as 30 ± 1 ppm. The initial total organic carbon of 4-tert-butylphenol (C_10_H_14_O, MW = 150.22 g mol^−1^, carbon present = 79.88% w/w) in the aqueous solution was measured via Total Organic Carbon analysis as 24.5 ± 0.5 mg L^−1^, which is close to the calculated theoretical value of 23.96 mg L^−1^. The stock solution was prepared on weekly basis and it was stored at 5.6 °C.

### Synthesis of Fe-doped TiO_2_ catalysts

Iron-doped TiO_2_ catalysts were synthesized with dopant concentrations of 0.5, 1, 2 and 4 wt.% using the wet impregnation method^65^. Specifically, 3 g of TiO_2_ (P25) were suspended in 100 ml of ultrapure water, and then the required amount of FeCl_2_ was added. The obtained mixture was constantly stirred for 24 hours and washed three times with distilled water to remove any precursor of physical adsorption before drying in an air oven at 80 °C for 12 hours. The dried solids were ground in a mortar and calcined at 500 °C for 6 hours in a muffle furnace.

### Characterization of Fe-doped TiO_2_ catalysts

The catalysts were characterized using X-ray powder diffraction (XRD, SmartLab automated multipurpose X-ray Diffractometer purchased from Rigaku), scanning electron microscopy (SEM, Auriga Cross Beam 540, Carl Zeiss) and transmission electron microscopy (TEM, JEM-2100 from Jeol Ltd., Japan equipped by EDS system, Inca Energy 350, Oxford Instruments PLC, UK). The XRD pattern was recorded using Cu Kα radiation, and the 2θ ranged from 10 to 80°. The average size of TiO_2_ nanoparticles was calculated based on the Scherrer equation^65^:

23 $$D=\frac{0.9\lambda }{\beta cos\theta }$$

where D is the crystallite size of the catalyst, λ is the X-ray wavelength (1.54060 Å), β is the full width at half maximum of the diffraction peak and θ is the diffraction angle.

The semi-quantitative (standard-less) chemical composition analysis of 4% Fe-TiO_2_ was carried out using X-ray fluorescence spectrometer (XRF, PANalytical Axios, UK). The sample weight 0.5 g and was prepared as homogeneous powder, which was analysed under He atmosphere.

In addition, UV-VIS spectroscopy analysis (Evolution 60 S UV-Visible spectrophotometer, Thermo Fisher Scientific) of all catalysts was carried out in the wavelength range from 200 to 750 nm. Prior to the UV-Vis analysis, the catalysts were sonicated in ethanol for 5 minutes.

### Reactor configuration and experimental procedure

**Figure 13:**
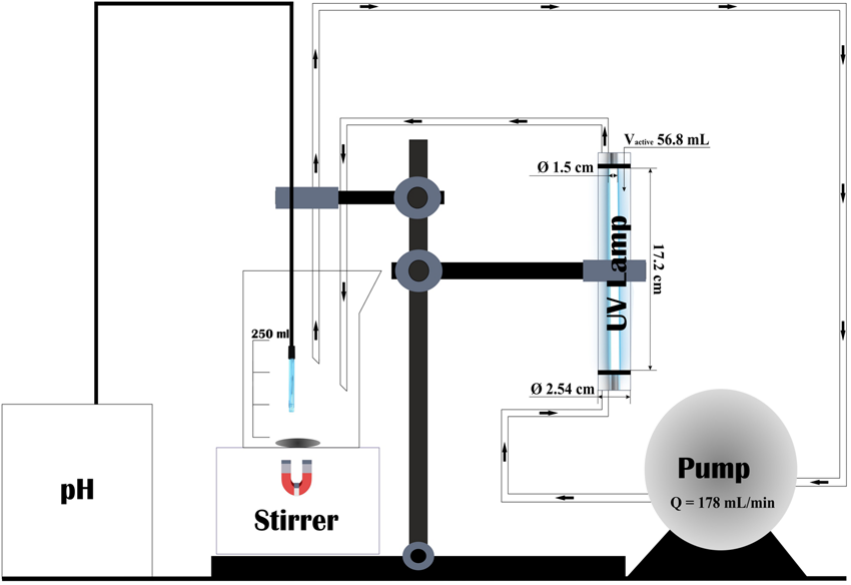
The experimental setup.

Experiments were carried out using the apparatus shown in Fig. 13. The total volume of the treated solution was 250 mL. An annular photoreactor of 56.8 mL irradiated volume, operated in batch recycle mode, was used. The solution was continuously pumped through the photoreactor at the rate of 178 mL min^−1^ using a peristaltic pump drive 5006 purchased from Heidolph. An Osram lamp with a power of 6 W, placed inside the photoreactor, emitted ultraviolet radiation of 254 nm. The non-irradiated part of the solution was constantly stirred with a magnetic stirrer (Bibby Scientific, United Kingdom). A Mettler Toledo LE409 electrode was immersed in the solution for continuous pH measurement.

TiO_2_ catalyst (P25) was used as base catalyst for both preparing doped photocatalysts and comparison purposes. Each separate experiment was repeated twice, and the average was used in plots using the standard error of the mean. The catalysts were sonicated in water for 10 minutes before use by means of a FB15055 ultrasonic bath from Fisher Scientific. Then, the aqueous solution containing the 4-tert-butylphenol with concentration 30 mg L^−1^ was mixed with the catalyst (1 g L^−1^) under constant magnetic stirring. Before UV irradiation, the solution was stirred for 1 hour in the dark so that the system reached adsorption equilibrium. The start of the UV lamp corresponded to zero time.

Each photocatalytic experiment lasted 60 minutes, and samples were periodically withdrawn and sent directly to analysis. A Vitlab 1000 μL automated pipette was used to take samples. 88.31 mg L^−1^ of H_2_O_2_ was used for the experiments on the effect of hydrogen peroxide addition on the photocatalytic performance.

### Analytical methods

The photocatalytic activity of the prepared catalysts was assessed via pH measurements, Total Organic Carbon (TOC), and High-Pressure Liquid Chromatography (HPLC) analysis. TOC analysis was performed using the Multi N/C 3100 instrument by Analytik Jena AG (Germany).

The 4-tert-butylphenol in the solution was quantified by HPLC Agilent 1290 Infinity II system.

The concentrations of acetic and formic acid in final solutions were determined using ion chromatography (IC) (930 Compact IC Flex supplied by Metrohm).

The iron content in the solution was determined using the atomic absorption spectrometer AAnalyst 400 from Perkin Elmer.

Prior to TOC and AAS analyses, all aqueous samples were filtered using Agilent Captiva premium syringe filters with a 0.45 µm regenerated cellulose (RC) membrane. For HPLC and IC analyses, the catalysts were separated from the solution by filtration through RC membranes with a pore size of 0.2 μm (Agilent Captiva premium syringe filters).

The removal efficiency was calculated according to Eq. (24):

24 $$Removal\,efficiency\,( \% )=(1-\frac{{C}_{t}}{{C}_{o}})\times 100$$

where *C*_*t*_ is the concentration after time *t* and *C*_o_ is the initial concentration.

## Conclusions

4-tert-butylphenol is an endocrine disruptor and considered as an emerging pollutant and a serious water contaminant because it persists in the environment, has acute and chronic toxicity, and estrogenic activity as well. In this study, Fe-doped TiO_2_ catalysts with different dopant concentrations (0.5, 1, 2 and 4 wt.%) were successfully prepared using the wet impregnation method, and their photocatalytic activities were tested in the degradation and mineralization of 4-tert-butylphenol in water under UV irradiation. The catalysts prepared were characterized via SEM, TEM, XRD and XRF, while their photocatalytic performance and stability was evaluated via TOC, HPLC, AAS, and IC analyses. The main conclusions are:

1. A well-distributed iron phase on the surface of TiO_2_ was observed for all Fe-doped TiO_2_ catalysts during SEM/EDS analysis.
2. 4-t-BP was rather easily degraded using UV/Fe/TiO_2_ or UV/H_2_O_2_ but lower TOC removals were achieved due to the formation of 4-t-BP degradation intermediates. The 4% Fe/TiO_2_ catalyst exhibited the highest carbon mineralization (86%) among the catalysts tested.
3. The concentration of all intermediates decreased while the carbon dioxide formed increased with increasing the iron content of the TiO_2_ catalysts.
4. The photocatalytic experimental results were well fitted by pseudo-second order kinetics. The reaction rate coefficients estimated ranged in 0.0064–0.0100 L mol^−1^ min^−1^.
5. 0.14 and 0.40 ppm of iron were detected in the solution after the UV/4%Fe/TiO_2_ photocatalytic treatment of 4-t-BP in the absence and presence of H_2_O_2_, respectively. Fe leaching was negligible taking into account the total iron load of catalysts.

## Data Availability

The datasets generated during and/or analysed during the current study are available from the corresponding author on reasonable request.
